# Direct and indirect effects of climate and seed dynamics on the breeding performance of a seed predator at the distribution edge

**DOI:** 10.1002/ece3.70104

**Published:** 2024-08-16

**Authors:** Daniel Oro, Lídia Freixas, Carme Bartrina, Silvia Míguez, Ignasi Torre

**Affiliations:** ^1^ Ecology and Complexity Department Center of Advanced Studies (CEAB‐CSIC) Blanes Spain; ^2^ BiBio Research Group Natural Sciences Museum of Granollers Granollers Spain; ^3^ Independent Researcher Barcelona Spain

**Keywords:** *Fagus sylvatica*, food availability, *Glis glis*, local adaptation, local meteorological conditions, marginal populations, Quercus spp, reproduction, seed production

## Abstract

Marginal populations usually have low densities and are considered to be particularly vulnerable to environmental stochasticity. Using data collected in nest boxes, we analyzed the breeding performance of the edible dormouse (*Glis glis*), an obligate hibernating rodent and a seed predator in deciduous forests, in two populations at the distribution range's edge. Despite being only 20 km apart from each other, Montseny is a large patch of mixed deciduous forests (oaks and beech), whereas Montnegre would be the harshest habitat, that is, a small, isolated patch with only oaks. First, we studied the differences in climate and tree cover change in the two populations. Second, we analyzed the direct and indirect roles of local climate conditions and seed availability on breeding performance over 10 years in each population. Finally, we explored the influence of tree cover change on the occupancy dynamics in the two populations. Our results showed contrasting responses between populations: in Montseny, asynchronous seed production between oaks and beech precluded skip breeding, and breeding performance increased with seed availability. Furthermore, dormice in Montseny may use pollen production to anticipate the amount of beech nut resources and adjust their breeding effort. Boxes showed higher occupancy and colonization and fewer extinctions in Montseny than in Montnegre, where seed availability did not drive breeding performance. Results from Montnegre suggest that skip breeding was an adaptive response to a more pulsed, harsher environment. Here, females produced a similar number of pups than at Montseny. Long‐term studies dealing with population responses in marginal habitats can lead to a deeper understanding of the capacities of organisms to adapt to harsh environments. Although local adaptation is frequently documented across various taxa, studies at the distribution edge may shed light on our still limited comprehension of the underlying mechanisms responsible for its occurrence.

## Graphical abstract

The study investigates the breeding performance of edible dormouse populations (*Glis glis*) at the distribution edge, focusing on Montseny (MS) and Montnegre (MN). Contrasting responses were observed, with MS showing increased breeding performance linked to seed availability and higher occupancy, while MN exhibited adaptive skip breeding in response to a harsher environment. Results suggest that MN dormice can adapt to challenging conditions, emphasizing the importance of understanding population responses in marginal habitats for comprehension of adaptive mechanisms.

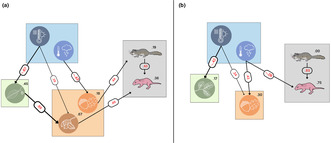

## 1 INTRODUCTION

Understanding the ecological dynamics of populations at the edge of their distribution is a multifaceted endeavor, offering a unique lens through which we can decipher the intricate processes shaping species endurance (Angert et al., 2020; Hardie & Hutchings, 2010; Usui et al., 2023). Exploring the responses to environmental changes of those populations has an additional interest since it is in the extremes that plasticity and adaptation are intensively acting to overcome the challenges of living in those habitats. Populations residing at the edges experience environmental conditions that tend to be harsher and less predictable than those faced by populations in the core of the distribution range (Pacifici et al., 2015, 2017). Furthermore, those populations frequently show lower densities, a consequence of the challenging environmental conditions and limited resource availability that occur in marginal habitats (Tranquillo et al., 2023). Lower densities make populations particularly susceptible to demographic stochasticity and the potential for local extinctions, rendering them more reliant on immigration from the core population. However, marginal populations may be highly fragmented, and local buffering mechanisms become crucial to overcome environmental stochasticity and more particularly the impacts of global change, such as climate warming and habitat transformation (Hampe & Petit, 2005; Nadeau & Urban, 2019; Usui et al., 2023).

Climate warming may be a driver of extinction in this century, through direct effects on species and synergies with other extinction agents, and together with changing landscapes – and associated pressures – may significantly reduce local terrestrial biodiversity (de Chazal & Rounsevell, 2009; Mantyka‐pringle et al., 2012). This seems to be the case in Mediterranean ecosystems, where land use and climate show high rates of change (Doblas‐Miranda et al., 2015; Myers et al., 2000; Valladares et al., 2014). For instance, new socioeconomic conditions, such as afforestation due to land abandonment in mountain areas increase forest cover and woodland communities (Pereira & Navarro, 2015; Ricotta et al., 2000). Changes in forest cover, driven by various anthropogenic and natural factors, may alter habitat structure and composition, thus driving resource availability and predator–prey dynamics (e.g. Orrock et al., 2003). On the other hand, the influence of climate on the fitness of individuals and population dynamics may be complex, especially for species above primary producers (Daskin & Pringle, 2016; Hamann et al., 2021; Menéndez et al., 2007). A representative of an intermediate trophic level is the edible dormouse (*Glis glis*), a seed consumer of deciduous trees. This rodent hibernates for an extended length of time – between 7 and 9 months – and shows much higher adult survival than rodents with similar body size (Lebl, Bieber, et al., 2011; Pilastro et al., 2003). Thus, life history theory predicts that dormice, compared with close phylogenetic species, should show lower and less variable reproductive success (Stearns, 1992). Additionally, we expect that dormice show anticipatory reproduction (i.e., reproduction decision is made before the pulse of the foraging resource; Vekhnik, 2019), which has also been reported in some rodent species such as pine squirrel (*Tamiasciurus hudsonicus*), red squirrel (*Sciurus vulgaris*) and eastern chipmunks (*Tamias striatus*; Bergeron et al., 2011; Boutin et al., 2013). Owing that dormice and species sharing life history tactics such as hibernation may tune their breeding effort to avoid survival costs of reproduction (for instance by skip breeding), evolutionary forces are selecting for identifying anticipatory environmental clues for decision making. Dormice serves as a compelling model to unravel the effects of rising temperatures, seed production, and forest cover changes on its breeding performance, especially at the edge of its distribution range. As a specialized seed predator, it needs to cope with the variable tree seed production (e.g., beech nuts and oaks) or masting pulsed resources (Lebl, Bieber, et al., 2011; Ruf & Bieber, 2020; Vekhnik, 2019), which is expected to be more stochastic in marginal habitats (Koenig & Knops, 2005; Pearse et al., 2016). Previous research suggests that rising temperatures may disrupt the synchronization between the breeding cycles and the availability of pulsed resources in several species, leading to shifts in reproductive phenology and potentially affecting their overall reproductive success (Bogdziewicz et al., 2020; Durant et al., 2007).

Here, we analyzed the breeding performance (i.e. annual number of breeding females and number of pups per breeding female) of two edible dormouse populations at the edge of their distribution range in the Mediterranean region, where they are potentially more exposed to environmental stochasticity and the impacts of global change (Pacifici et al., 2015, p. 201; Sexton et al., 2009). The two areas, Montseny and Montnegre, are only 20 km apart but exhibit distinct ecological features. Montseny closely matches the environmental conditions typical of core dormouse habitats, particularly in terms of deciduous forests and climate. In contrast, the dormouse population in Montnegre occupies a more challenging habitat, a small and isolated patch at a lower altitude with a more Mediterranean climate, characterized by drier and warmer conditions. First, we compared the patterns of climate and landscape changes in each population to corroborate that the environmental conditions are different between populations. Second, we analyzed the direct and indirect roles of local climate conditions and seed availability on breeding performance over 10 years. Finally, we explored the influence of tree cover change on the occupancy dynamics of nest boxes in the two populations. We expected that all the study parameters would show lower values for the more stressed population (Montnegre) and that those parameters would be lower than those recorded in the populations studied at the core of their distribution. For instance, in years of poor seed production in the populations of the core distribution, where the unique seeds present are beech nuts, there are no signs of sexual activity in females and males (Bieber, 1998; Schlund et al., 2002). Skip breeding, where medium‐ and long‐lived species forgo reproduction during years with harsh environmental conditions, serves as an adaptive strategy to conserve energy and resources, thereby enhancing long‐term adult survival and reproductive success over an individual's lifespan (e.g., Oro et al., 2014). However, nothing is known about sabbatical years in marginal Mediterranean populations. By examining the effects of climate, seed production, and forest cover changes on breeding performance and nest occupancy, we aim to contribute to a deeper understanding of the adaptive strategies employed by species facing environmental shifts at the periphery of their range.

## 2 MATERIALS AND METHODS

### 2.1 Study species and areas

**FIGURE 1:**
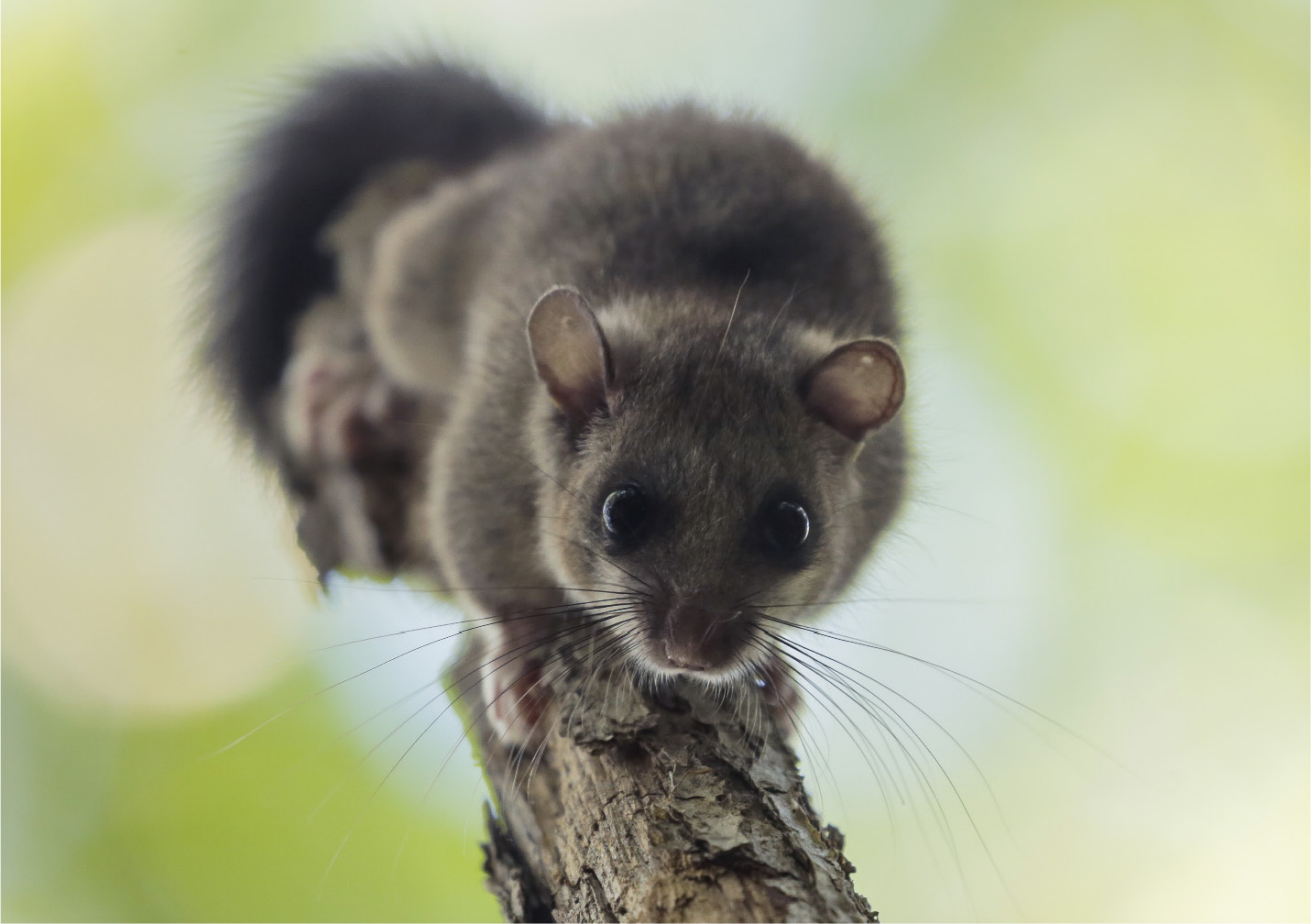
Adult edible dormouse (*Glis glis*) in the Montseny Natural Park (Catalonia, Spain). Author: Oriol Massana.

Edible dormice (*Glis glis*) are arboreal, nocturnal, and obligate hibernating rodents (Figure 1). Its populations in the Northern Iberian Peninsula mate during July and August, and newborns occur between August and September of the same breeding year (Freixas et al., 2011). Therefore, the lactation period and juvenile phase coincide with the time of higher food availability, which is essential to win body fat reserves within a short period to survive their first hibernation season (Bieber & Ruf, 2009; Lebl, Rotter, et al., 2011).

**FIGURE 2:**
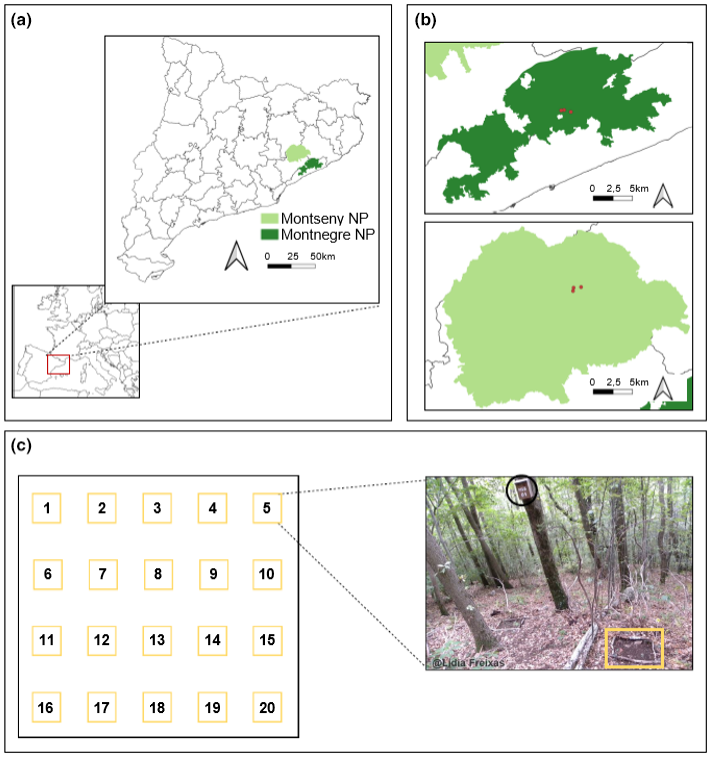
(a) Location of the two study sites, Montseny and Montnegre Natural Parks. (b) The six plots in red. Distances between plots (centroids of the plot) were 360, 650, and 940 m in Montseny and 320, 795 and 1100 m in Montnegre. (c) Distribution of the 20 nest boxes in each plot, which includes seed data grids. The picture shows a nest box (black circle) and the seed sampling grid in yellow. The maps were created with QGIS (QGIS Development Team, 2023).

The study sites are located on two different natural reserves – Montseny and Montnegre – in Barcelona province (Catalonia, NE Spain, Figure 2).

Both areas have Mediterranean climate and vegetation. A Mediterranean climate, characterized by hot, dry summers and mild, wet winters, contrasts sharply with the temperate climate in core populations of dormice in central Europe, which experiences more evenly distributed precipitation throughout the year and cooler summer temperatures, resulting in distinct ecological adaptations in each region. Nevertheless, they also show differences in the surface of suitable habitat and likely in climate (Bolòs, 1983). Montseny (41°48′29.41″ N, 2°22′3.18″ E) is a massif of about 400 km^2^ characterized by its moderate elevation (1700 m above sea level, m.a.s.l. hereafter). The study sites are located at an altitude between 1078 and 1143 m.a.s.l., and the vegetation is characterized by its heterogeneous composition, with *Quercus petraea*, *Fagus sylvatica*, *Corylus avellana*, and *Acer opalus*, mixed with *Quercus ilex* and *Ilex aquifolium* and surrounded by beech‐dominated forest (Ferrandiz‐Rovira et al., 2016). Montnegre range (41°39′37.7″ N, 2°33′4.43″ E) is smaller (ca. 150 km^2^) and the culminating ridge is a ca. 3 km long above 700 m.a.s.l. (Panareda & Salvà, 2001), where the study plots are located (Figure 2). Vegetation is composed of *Q. canariensis, Q. petraea, Q. ilex and C. avellana, Castanea sativa, mixed with I. aquifolium and Prunus avium*, with some isolated trees of *F. sylvatica* (Ferrandiz‐Rovira et al., 2016). We assumed (see also results on climate and land‐use changes below) that the environment was harsher in the Montnegre than in the Montseny. We anticipate that Montnegre climate will be more Mediterranean (drier and warmer), where land‐use changes (i.e. the deciduous forest cover dynamics) will have a greater impact due to the limited availability of suitable breeding habitats.

### 2.2 Data collection

#### 2.2.1 Meteorological and landscape data

Meteorological local data were provided by the closest two meteorological stations to the study plots, called “Viladrau” (953 m.a.s.l.) and “Pla de la Tanyada” (690 m.a.s.l.). “Viladrau” is located at a straight‐line mean distance of 5.5 km from the three study plots in Montseny, while “Pla de la Tanyada” is located at a straight‐line mean distance of 0.6 km from the three study plots in Montnegre. Data from both stations included daily mean, maximum, and minimum temperatures as well as cumulative rainfall values. The temperatures and precipitation were calculated on a seasonal basis: spring (mean of April, May, June) and summer (mean of July, August, September).

Official land cover maps were used to estimate the change in forest cover of deciduous and sclerophyllous trees (seed producers) and pines between 2007 and 2017 (González‐Guerrero & Pons, 2020), both around plots and at the elevation belt where plots were situated (i.e., 600–800 m.a.s.l. in Montnegre, and 1000–1200 m.a.s.l. in Montseny). Despite, the period did not match exactly the period of data recorded in the present study (2012–2021), we expected that landscape change would show the same patterns and trends described in Catalonia (Ameztegui et al., 2016). We used the 1 km^2^‐UTM resolution, owing to that there was autocorrelation between land cover changes at different spatial scales (e.g., 100, 500, and 1000 m‐buffers; Baśnou et al., 2013; Torre & Díaz, 2022).

#### 2.2.2 Dormouse data

The edible dormouse data were collected by the Dormouse Project (www.dormice.org) conducted by the Natural Sciences Museum of Granollers. This project has been active from 2004 until the present, with almost 50 stations active around Catalonia and 20 in Germany. Nevertheless, in this study, we used data from 2012 to 2021 from one of the southernmost Iberian populations, located in Montseny and Montnegre Natural Parks. We considered the number of breeding females and pups for the analysis. We used breeding females and not males because we are evaluating the effects of climate and fruit availability on dormice reproductive performance. We assumed that females are the ones who decide whether or not to reproduce depending on environmental conditions and they are in total charge of parental care. Dormice are polygynous, that is, a female can mate with more than one male. After mating, males disperse and stay outside the territories of the females. For pups, we use data related to the first three age groups (i.e., pink pups, gray pups, and open‐eyes pups; Vekhnik, 2019). To collect these data, we used the method of capture–mark–recapture on six sampling plots of nest boxes (30 × 15 × 15 cm, with a 5‐cm entry hole) attached to trees at a height of ~3 m. Nest boxes were set 25–30 m apart on a grid of 5 × 4 nest boxes, with 20 boxes per plot site (Torre et al., 2021), covering a minimum area of 1 hectare each (see Figure 2b). Nest boxes are used by 90% of the breeding population during the breeding years, but only by 35% of individuals known to be alive during the non‐breeding years (Trout et al., 2015). The nest boxes were used by edible dormouse during the active season, and they were checked, during the day, 10 times each year. These were performed every 2 weeks, from mid‐July to mid‐November. Other checks were performed before the breeding period, when edible dormouse is non‐active inside the boxes, to repair and clean the nest boxes. If there was an individual inside the nest boxes, they were taken inside a textile bag, identified by a unique number, weighed, sexed, and checked their reproductive condition. Females were considered sexually active if they had visible mamma or were found inside a nest box with pups. Depending on their age, the weight of pups ranges from 2.7 to 39.8 g (Míguez et al., 2023). The pups were weighed, aged, and sexed. Finally, adults (after second hibernation), yearlings (after first hibernation, already sexually active), and juveniles (before the first hibernation), were marked using a transponder (AVID Musicc, 8 × 2.1 mm) injected into the skin of the neck to make an easier identification or numbered with a metal ear‐tag (Style 1005–1, National Band and Tag Co., USA) placed on the ear (Ferrandiz‐Rovira et al., 2016; Freixas et al., 2011). Research on live animals followed ethical guidelines (Sikes and Animal Care and Use Committee of the American Society of Mammalogists, 2016), the project was approved, and captures were performed under the special permission of the Catalan Government (Generalitat de Catalunya).

#### 2.2.3 Seed availability and pollen data

Seed availability was estimated from years 2012 to 2021, by counting one at a time all the seeds – intact and predated by rodents or insects– that were found within 20 sampling grids on the ground (50 × 50 cm) placed under each nest box (Figure 2c; used as a proxy of seed production; Puig‐Gironès et al., 2023). In the study areas, we found up to four seeds: acorns, beech nuts, hazelnuts, and chestnuts. We only considered acorns and beech nuts since they were the commonest seeds in Montnegre and Montseny (98.4% and 99.9% of the total number of seeds counted in the study, see also Table S3). Seed counting was conducted every 15 days from the end of August to the end of November, resulting in a total of six sampling days each year in the two study areas. To prevent double counting, the sampling squares were cleaned after each check. Only sound seeds (with apparent viability for being dispersed and/or germinated) were considered owing that both sound and infested/predated seeds showed a strong correlation (acorns: *r =* .76; beech nuts: *r =* .92). Furthermore, total seed count was strongly correlated with total weight for both acorns and beech nuts (*r* = .96 and *r* = .98, respectively, *p* < .0001 both), and only the first variable was considered for the analyses. Pollen data were provided by the Catalan Aerobiological Network (Xarxa Aerobiològica de Catalunya, XAC). Data have been obtained following international standards (Galán et al., 2014). We selected *Quercus* and *Fagus* pollen types for the study period (2012–2021). For the analysis, we used the mean of the annual pollen integral (APIn) from three stations close to the study area, all within a mean radius of 46 km: Bellaterra, Girona, and Manresa.

### 2.3 Data analysis

We analyzed spatial and interannual differences in seed availability and breeding performance using generalized linear mixed models (GLMM), including the plot as a random factor. Spatial variations in climate (mean temperature and cumulative rainfall) were analyzed with general linear models (GLM). Models showing dispersion irregularities (underdispersion, with scaled deviance <0.5 or overdispersion, with scaled deviance >1.5) were built with negative binomial (*glmmTMB*; Brooks et al., 2017) instead of Poisson (*lme4*; Bates et al., 2015) error distribution to adapt better the models to the data (Zeileis et al., 2008). For the selected models, we calculated pseudo‐*R* ^2^ values (Nakagawa & Schielzeth, 2013) using the R function *r.squaredGLMM* and the delta method for variance estimation. GLMMs were conducted using R software version 4.1.2 (R Core Team, 2012).

The association between local weather conditions, pollen production, seed availability, and dormice breeding, was assessed by structural equation modeling (SEM), an extension of multiple regression which was developed to decompose correlations into different pieces for interpretation of direct and indirect effects (Everitt & Dunn, 1991). The rationale for using SEM instead of conventional regression was that temperature and precipitation may have both direct (mediated by physiological demands) and indirect (mediated by the effects on pollen and seed production) effects on dormice populations. To avoid multicollinearity of meteorological variables we used principal components analysis (PCA) to obtain orthogonal components that were interpreted as gradients of meteorological variables for spring and summer data (Dytham, 2011). We tested the significance of regression paths by means of Amos software (version 5.0, James L. Arbuckle 1983–2003). The appropriateness of models was rated by means of a stepwise selection procedure using the Bayes Information Criterion (BIC), which assigned a greater penalty to model complexity, and therefore, picked the most parsimonious models. A matrix permutation test (using 500 permutations) was used to assess whether the selected models fit better than completely randomized models.

Multiple‐season single‐species occupancy models were used to analyze dormice demography (MacKenzie, 2006). This approach uses two separate processes, which are subject to a hierarchical structure for modeling the “state process,” dealing with the species distribution and its drivers, and the “observation process,” dealing with data collection. The timeframe database considers two temporal scales: first, a short one, consisting of the detection/non‐detection of the species (i.e., breeding females) during six consecutive nest box checks conducted along the breeding season to account for detectability. Second, a longer period consisting of the detection/non‐detection of the species during the six cumulative surveys conducted within a year (primary occasions) to account for changes in occupancy and the rest of the parameters between years. The parameters calculated for breeding females were occupancy (*ψ*), that is, the proportion of boxes that are occupied; colonization (*γ*), that is, the probability that an unoccupied box in season *t* is occupied in season *t* + 1; extinction (*ε*), that is, the probability that a box occupied in season *t* is unoccupied in season *t* + 1; and detectability (*p*), that is, the probability of detecting breeding females when present. Competing occupancy models were fitted with the software PRESENCE (Mackenzie, 2012), starting with a null model (setting all parameters constant), considering that occupancy rates and derived parameters did not change in space and time [*ψ*(·), *γ*(·), *ε*(·), *p*(·)]. The models were adjusted by adding vegetation composition profiles surrounding the trapping plots 5 years before the start of the study (2007 cover of deciduous trees as a site‐dependent covariate) and time effects. Alternative models were fitted considering that significant land‐use changes (between 2007 and 2017) were observed at the landscape level considering that parameters could be affected by land‐use change at a spatial scale larger than the limits of the sampling plots. Since the cover of deciduous and sclerophyllous trees was inversely correlated within plots (*r* = −.99 for both years 2007 and 2017), we only included the cover of deciduous trees in the models to avoid redundancy. To ascertain whether changes in landscape covers were consistent among study areas, elevation belts, and plots between 2007 and 2017, Wilcoxon matched‐pairs tests were used. Mean and standard deviations were used as descriptive statistics unless otherwise stated.

## 3 RESULTS

### 3.1 Patterns of climate and landscape change

Along the study period (2012–2021), the Montnegre plots showed statistically significant higher mean annual temperature ($\bar{x}$ = 12.87°C ± 0.31) and lower cumulative rainfall ($\bar{x}$ = 537.36 mm ± 182.78) than the Montseny plots ($\bar{x}$ = 11.15°C ± 0.35; $\bar{x}$ = 988.94 mm ± 347.43: *F*_1,9_ = 101.30, *p* < .0001 and *F*_1,9_ = 20.44, *p* < .001, respectively). Mean temperature increased along the study period by +0.6°C in the Montnegre (*r* = .65, *p* = .04, *n* = 10), but remained invariable in Montseny (*r* = −.04, *p* = .91, *n* = 10; Table S1). Cumulative rainfall did not show any temporal trend in both areas (*r* = .30, *p* = .42, *n* = 10 and *r* = .04, *p* = .86, *n* = 10, respectively).

The Montnegre experienced strong landscape changes within 10 years (2007–2017), with a decline of sclerophyllous (1944 ha lost, −15.78% of total cover, *z* = 8.35, *p* < .0001, *n* = 227) and deciduous forests (506 ha lost, −33.59% of cover, *z* = 6.49, *p* < .0001, *n* = 227), at the expense of the expansion of pine forests (2980 ha gained, +64.0% of cover, *z* = 11.79, *p* < .0001, *n* = 227). Interestingly, in the Montseny, a contrary pattern was observed, with a decline of 600 ha in forest cover. Despite deciduous forest declined (744 ha lost, −7.90% of cover, *z* = 4.05, *p* < .0001, *n* = 249), sclerophyllous forests increased (1024 ha gained, +6.85% of cover, *z* = 5.25, *p* < .0001, *n* = 249), in that case at the expense of pine forests (892 ha lost, −14.46% of cover, *z* = 4.63, *p* < .0001, *n* = 249; Figure S1).

**FIGURE 3:**
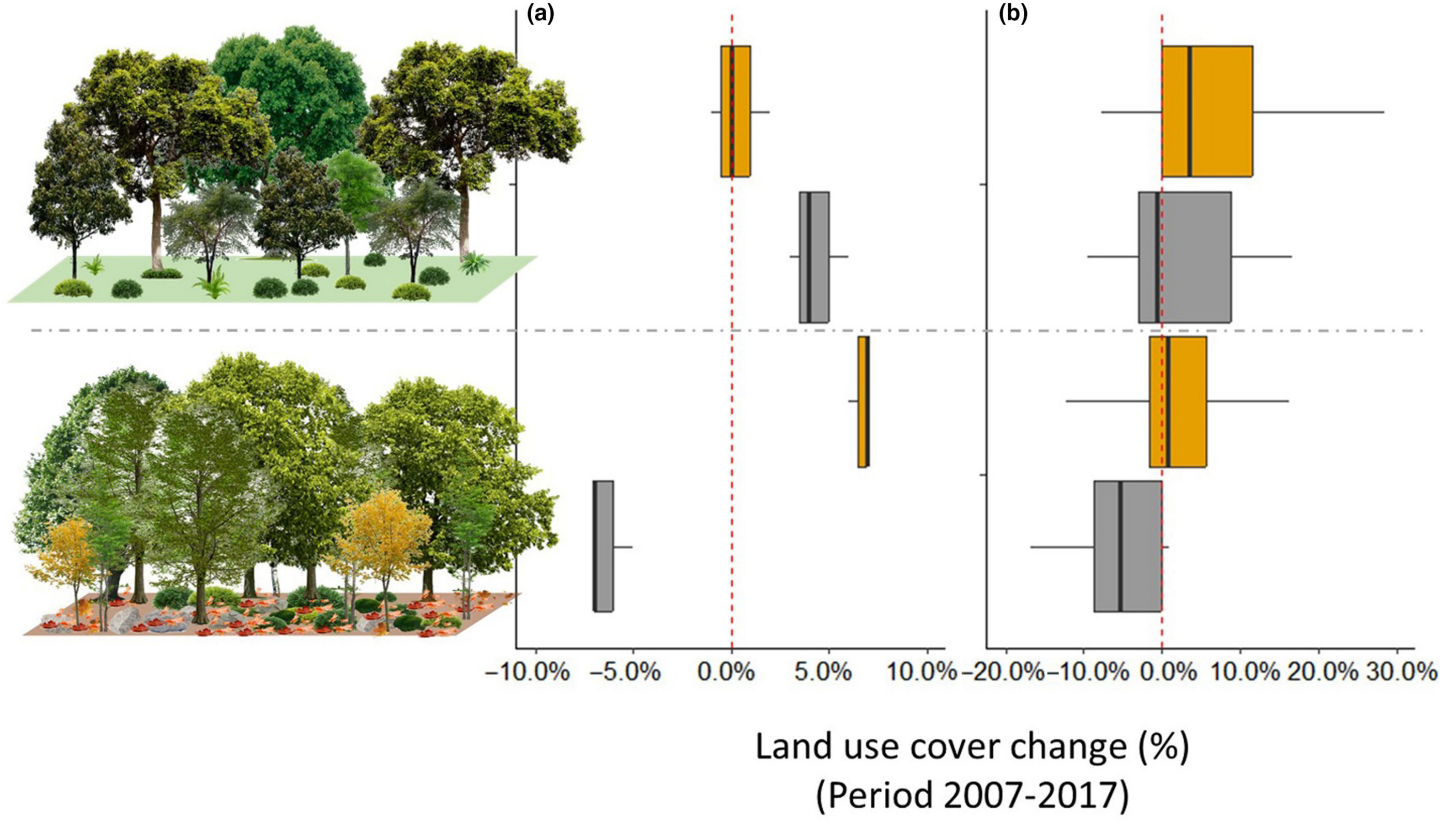
Boxplots of land cover changes occurred between 2007 and 2017 (in %) in sclerophyllous (upper panel) and deciduous forests (lower panel) around (a) the sampling plots (1 km^2^) and (b) within the elevation belts where the plots were placed at Montseny (elevation: 1000–1200 m, orange boxes) and Montnegre (elevation: 600–800 m, gray boxes; see also Figure S1).

We confirmed a significant decline in the cover of deciduous forests in the UTMs within the elevation belt where the plots were situated at MN (600–800 m.a.s.l.: *z* = −2.8, *p* < .01, *n* = 10, $\bar{x}$ = −5% per km^2^), but this was not observed at Montseny (1000–1200 m.a.s.l.: *z* = 1.50, *p* = .13, *n* = 55, $\bar{x}$ = +1.3%, Figures 3 and S1). Otherwise, we confirmed a statistically significant increase in the cover of sclerophyllous forests in Montseny (1000–1200 m.a.s.l.: *z* = 4.54, *p* < .0001, *n* = 55, $\bar{x}$ = +7.7%), but this was not observed at Montnegre (600–800 m.a.s.l.: *z* = 0.86, *p* = .38, *n* = 10, $\bar{x}$ = +6.7%). In this latter case, despite the overall mean change seeming high, there was heterogeneity since five UTMs showed an increase and the other five a decline.

At the plot level, we observed significant differences in forest composition between both study areas. In Montseny, the three plots were almost surrounded by deciduous forests (92% of the area covered) and in Montnegre they were mostly surrounded by evergreen broad‐leaved forests (60%). Deciduous forest only covered 26% of the area in this latter case. Land cover change in the period 2007–2017 (Figure 3), with an increase in deciduous forests in Montseny ($\bar{x}$ = +6.6% cover per km^2^), and a decrease in Montnegre ($\bar{x}$ = −6.3% cover per km^2^). On the other hand, we observed the stability of broad‐leaved sclerophyllous forests in Montseny ($\bar{x}$ = −0.33% cover per km^2^) and an increase in Montnegre ($\bar{x}$ = +4.6% cover per km^2^).

### 3.2 Patterns of seed availability

**FIGURE 4:**
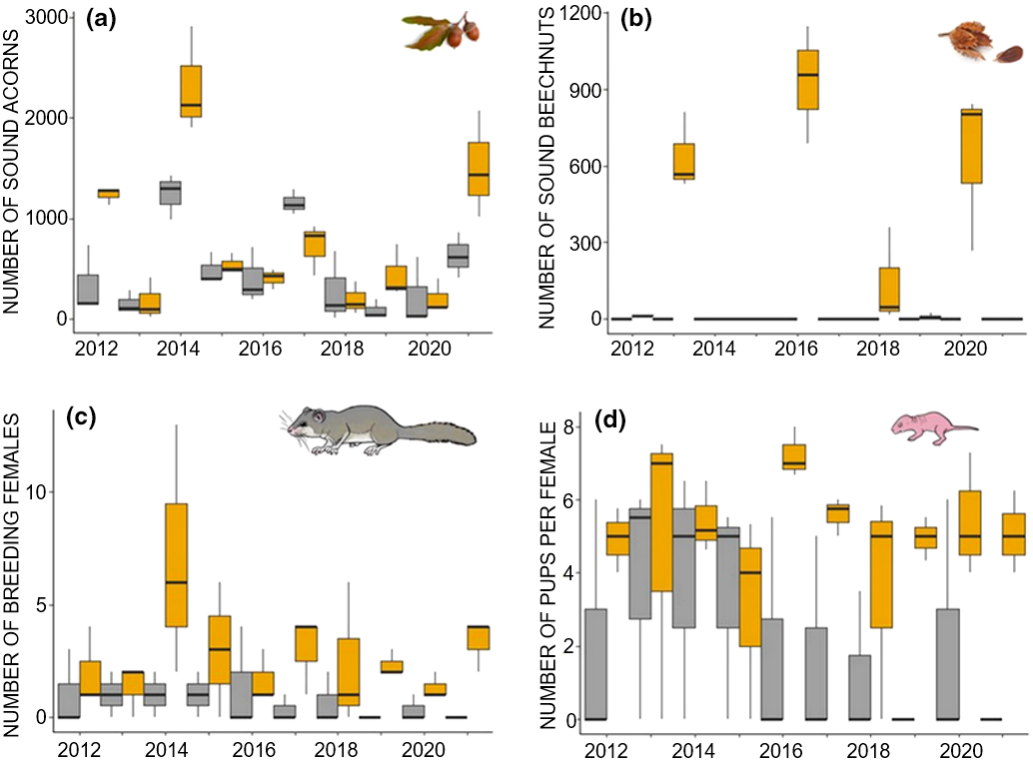
Median (±quartiles) of (a) number of sound acorns, (b) number of sound beech nuts, (c) number of breeding females, and (d) number of pups per female, along the study period (2012–2021) in the three sampling plots placed at Montseny (orange color) and Montnegre (gray color) Natural parks.

Both study sites showed variable seed availability over the years. Several species of oaks (*Q. ilex, Q. canariensis, Q. petraea*) and beech were considered the main seed producers, along with other occasional seeds like hazelnuts or ash seeds. Oak availability was steadier than beech, and it showed higher mean values in Montseny than in Montnegre, with a significant interaction between year and locality (Figure 4a, Table S2). Beech was only available in certain masting years (Figure 4b, Table S3). Moreover, there was a negative association between the availability of acorns and beech nuts (Montseny: *r* = −.47, *p* < .05, *n* = 30). The proportion of sound seeds was similar for acorns ($\bar{x}$ = 67.60% ± 10.59) and beech nuts ($\bar{x}$ = 71.06% ± 13.38).

### 3.3 Drivers of breeding performance and occupancy dynamics

In the Montseny, a total of 85 reproductive females of edible dormice were captured during the study period, with a maximum of 24 lactating females inside the nest boxes in 2014 (Figure 4c and Table S3). Here, 441 pups were also counted in nest boxes, with a maximum of 104 pups in 2014, and the highest median values in 2013 and 2016 (Figure 4d). In the Montnegre, a total of 20 reproductive females of edible dormice were captured during the study period, with a maximum of four lactating females inside the nest boxes in 2016 (Figure 4c and Table S3). Additionally, 109 pups were counted in nest boxes in that area, with a maximum of 22 pups in 2016 (Figure 4d).

On average, the Montseny plots showed four times more breeding females than the Montnegre (2.73 ± 0.53, and 0.67 ± 0.53 respectively), with a maximum of 14 and 4 individuals/plot, respectively (Figure 4c, Tables S2 and S3). However, the number of pups per breeding female was similar at both sites (5.58 ± 1.15 and 5.41 ± 0.80 for Montseny and Montnegre, respectively, Table S3).

**FIGURE 5:**
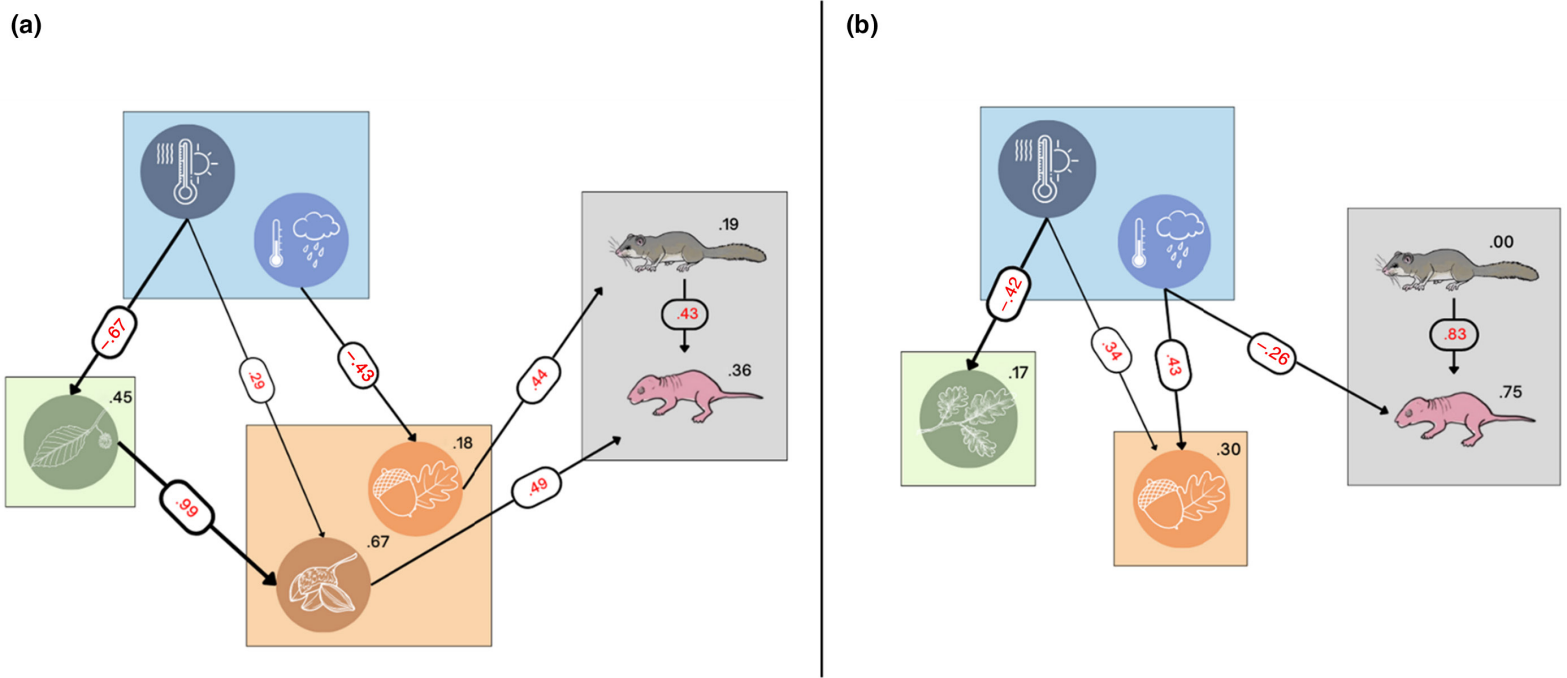
Structural equation models (SEM) showing the significant paths between climatic factors (blue square: Dark blue circle–Spring PC1, and light blue circle–Summer PC1), pollen (green square), seed counts (orange square: Orange circle–acorns, and brown circle–beech nuts), and the dormice breeding females and pups, at Montseny (a) and Montnegre (b). All significant regression coefficients showed inside ellipses, and *R* ^2^ explained by all predictors showed inside squares. The thickness of the lines indicates stronger associations.

The first SEM (Montseny) fitted a total of 112 models (out of 4096) that were ordered according to the BIC criterion (Table S4). The most parsimonious model showed seven significant paths and high goodness of fit (only three randomized models out of 500 showed best fit, *p* = 3/500 = .006). The model selected, which included spring and summer weather conditions of the same year, showed that these variables influenced the pollen and seed availability of both beech and acorn. Spring weather conditions (summarized by one PC positively associated with temperature and negatively with rainfall) showed a strong and negative effect on beech pollen production, but a small positive effect on beech availability. This means that beech pollen production was greater in cold and wet springs, but beech availability was favored in warm and dry springs. Furthermore, beech pollen production was strongly associated with beech availability (*r* = .99, *p* < .0001), but acorn pollen was not associated with acorn availability (*r* = .29, *p* = .11). Summer weather conditions (summarized by one PC also showing a positive association with temperature and negative to rainfall) showed a negative effect on acorn availability, but no effect on pollen production was detected (Figure 5a and Table S4). It was also found a relationship between edible dormouse reproductive females and acorn seed availability (Figures 4a,c and 5a), and between beech nuts availability and the ratio of pups per female (Figures 4b,d and 5a), but no direct effects of weather on either breeding females or pups per female were detected.

The second SEM (Montnegre) fitted a total of 92 models (out of 1024) that were ordered according to the BIC criterion (Table S5). The most parsimonious model showed four significant paths and high goodness of fit (only two randomized models out of 500 showed best fit, *p* = 2/500 = .004). The model selected, which included spring and summer weather conditions of the same year, showed that some of those influenced the pollen and acorn availability. Spring weather conditions (summarized by one PC positively associated with temperature and negatively with rainfall) showed a negative effect on *Quercus* spp. pollen production, and a positive effect on acorn availability. This means that *Quercus* spp. pollen production was greater in wet and cold springs, but acorn availability increased in dry and warm springs. But *Quercus* spp. pollen was not associated with acorn availability (*r* = .23, *p* > .05). Summer weather conditions (summarized by one PC also showing a positive association with temperature and negative to rainfall) showed a positive effect on acorn availability, but no effect on pollen production was detected (Figure 5b and Table S5). Surprisingly, no relationships between acorn availability and edible dormouse reproductive females or the ratio of pups per female were detected (Figure 5b). Nonetheless, summer weather conditions (i.e., warm and dry summers) in Montnegre negatively affected the breeding output (number of pups per female; Table S5).

**FIGURE 6:**
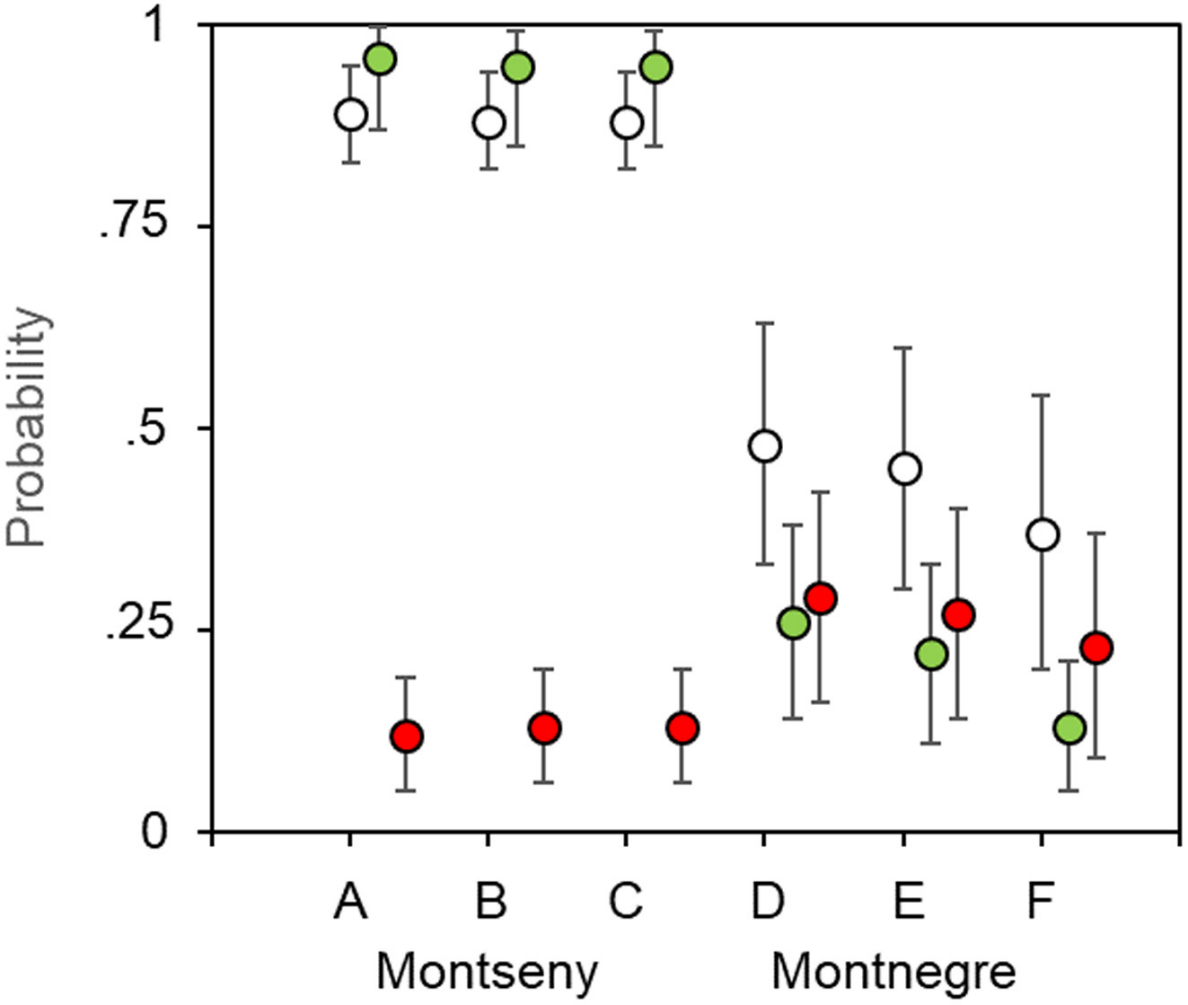
Probability (±SE) of occupancy (white dots), colonization (green dots), and extinction (red dots), for the dormice breeding females monitored for 10 consecutive years in six plots at Montseny (A–C) and Montnegre (D–F), after the second selected occupancy model [*ψ*(cover) *γ*(cover) *p*(cover)]. See Table S6 for details.

Multiple‐season occupancy models showed that the occurrence of breeding females in nest boxes was positively affected by the cover of deciduous trees around the sampling plots (Table S6). Only two models showed delta‐AIC values <2, the first selected the deciduous tree cover for the occupancy (27% of support), and the second selected the deciduous tree cover for occupancy, colonization, and detectability (16% of support, Figure 6). This means that the three calculated parameters increased with the cover of deciduous trees around the plots, but the derived parameter (extinction) showed an opposite pattern. Some other models were very close to the threshold of significance, such as the third one (10% of support; Table S6), which included the cover for occupancy, and the cover change of deciduous trees for colonization. This means that the probability of colonization was associated with the overall deciduous cover, but also was related to the cover change. Overall occupancy of breeding females was twice as large in Montseny than in Montnegre (88% vs. 43%), but the colonization probability was five times lower at Montnegre (20% vs. 95%). Extinction probability was twice as large in Montnegre than in Montseny (26% vs. 13%). Despite detectability being higher in Montseny (*p* = .45, SE: 0.04), the difference with Montnegre (*p* = .33, SE: 0.06) was not so large to produce significant biases in occupancy and the other parameter estimates.

## 4 DISCUSSION

In this study, we analyzed the effects of two agents of global change (i.e., climate and landscape changes) on the breeding performance of two marginal populations of edible dormice. A detailed long‐term study on seed availability allowed us to study its effects on the breeding performance of dormice in interaction with climate variability in two close but distinct habitats at the edge of the species distribution. Food and seed availability and climate are crucial drivers for survival and reproduction in rodents and small mammals including hibernating species (Bronson, 2009; Findlay‐Robinson et al., 2023; Le Coeur et al., 2022). We found complex and differential responses of dormice reproduction between the two populations, which suggest first, that local environmental conditions may drive local ecological responses, and second that those conditions may impose differential selection for adaptation in peripheral populations exposed to extreme and stochastic biotic and abiotic stressors (Angert et al., 2020; Hardie & Hutchings, 2010; Usui et al., 2023). For instance, the Montseny population, which is closer to environmental conditions in the core populations (by climate and the availability of both oaks and beeches), also shows a “sit‐tight strategy” described in central Europe, where dormice wait for optimal environmental conditions, specifically energy‐rich seed pulses, before reproducing (Bieber & Ruf, 2009; Kager & Fietz, 2009). In Montnegre, where seed abundance is much lower and the climate is drier and warmer, the number of annual breeding females was more constant than in Montseny, whereas the contrary occurred for their breeding performance (i.e., litter size was more variable in the former, harsher patch). In core habitats where populations may occupy patches with either low or high seed abundance, dormice in the former patches show lower reproductive investment and increased longevity, as a strategy to maximize their lifetime reproductive success (Bieber & Ruf, 2009).

### 4.1 Patterns of environmental dynamics at each population

Despite the two study populations being at the edge of the distribution and close to each other (only ca. 20 km), results confirmed that environmental conditions at Montnegre were harsher than at Montseny. Climate differences between the two areas were driven by differences in elevation (much higher in Montseny reaching up to 1706 m), topography (rougher slopes in Montseny), and proximity to the sea; being closer to the coast and at a lower elevation, Montnegre experiences higher and more stable temperatures than Montseny. Although total rainfall did not show any temporal trend in both areas, Montnegre exhibited a notable increase in mean annual temperature over the study period. Concurrently, landscape alterations in Montnegre showcased a shift towards pine‐dominated forests, contrasting the decline of deciduous and sclerophyllous forests. In contrast, Montseny experienced stability in temperature and rainfall, with an increase in sclerophyllous forests. Seed availability, a crucial determinant for the breeding success of seed predators, was higher in Montseny, which suggests a potential resource advantage in this population. Here, the negative annual association between acorn and beech nut availability underscores the intricate dynamics of seed availability, with potential cascading effects on the edible dormouse's breeding success (Bogdziewicz et al., 2020; Koenig & Knops, 2000, 2005; LaMontagne et al., 2020). Highly variable and asynchronous seed production has been described for several seed producers of the tree community in the study region and other temperate regions of the planet (Espelta et al., 2008; Fernández‐Martínez et al., 2012; Isagi et al., 1997; Koenig et al., 2010).

Our results supported that seed availability of oaks and beech was affected by local weather conditions (Bogdziewicz et al., 2019; Nussbaumer et al., 2018; Pearse et al., 2016; Piovesan & Adams, 2001). We found a significant relationship between seed production with summer conditions, both temperature and rainfall, which could be due to the importance of those conditions for pollination and seed growth (Bogdziewicz et al., 2017, 2019; Espelta et al., 2008; Koenig et al., 2010). The correlation between annual pollen and beech nuts availability (see also Bogdziewicz et al., 2017; Kasprzyk et al., 2014; Koenig & Knops, 2000) was strong and confirmed that beech seed production, almost absent in Montnegre and relatively abundant in Montseny, could be forecasted by dormice on pollen production. Other studies also dealing with boom and bust cycles of seed availability in pulsed systems show that predatory small mammals may anticipate the amount of foraging resources and adjust their breeding effort, whereas adult survival remains stable (Bergeron et al., 2011; Boutin et al., 2013; Clark et al., 2019; Dri et al., 2022). In contrast, this relationship between pollen and seed production was not significant for oaks, for which acorn availability is independent of pollen production (Fernández‐Martínez et al., 2012). This could be a consequence of the heterogeneity of pollen produced in space and time by the different tree species within the genus *Quercus* (*Q. petrea, Q. canariensis, Q. ilex*), which showed a differential species composition in each study area.

Those differential environmental trajectories in each study population highlight their ecological uniqueness, corroborating the premise that edge populations often face harsher conditions and higher spatial heterogeneity in environmental stochasticity (Usui et al., 2023). However, breeding performance, which was estimated using mean annual litter size, was similar in our study populations than in core populations, which are on average 5–7 pups per breeding female (Kager & Fietz, 2009; Lebl et al., 2010; Pilastro et al., 2003). Variance in the annual litter size of dormice in Montseny and Montnegre is also close to what is recorded in studies at the core areas of the distribution range (Kager & Fietz, 2009; Lebl et al., 2010). In contrast, the variance in the number of annual breeding females recorded in nest boxes was much larger in Montseny than in Montnegre, which suggests that lower food availability may result in a lower individual heterogeneity of life history tactics within populations (Bieber & Ruf, 2009). Furthermore, asynchronous seed production between oaks and beech in Montseny reduced skip breeding, which seems to be a more common strategy for dormice inhabiting forests dominated by single tree species, such as beeches in some core areas and deciduous oaks in Montnegre (Lebl et al., 2010; Pilastro et al., 2003; Ruf et al., 2006). Studies in other edge dormice populations suggest that dormice may adapt to very different forests described for habitats in the core areas. For instance, other Mediterranean dormice populations prefer mixed evergreen forests of cork and holm oaks to forests dominated by beeches (Marteau & Sarà, 2015), while dormice inhabiting the eastern edge forests outside of beech distribution feed on a diverse diet composed of acorns, hazelnuts, birch seeds, and mushrooms (Vekhnik, 2019). All in all, we were unable to assess whether our study population showed a trade‐off between reproductive performance, skip breeding, and survival that could enhance fitness prospects and support stable population growth rates at the edge of the distribution range. Nevertheless, we expect that survival rates in our study populations will be lower than those in core habitats, as longer hibernation periods associated with lower temperatures typically enhance survival (Elvert & Heldmaier, 2005; Evans et al., 2023; Giroud et al., 2023; Hoelzl et al., 2015; Lebl, Bieber, et al., 2011; Ruf & Bieber, 2020). Our results highlight the importance of spatial scale and environmental heterogeneity for interpreting and identifying ecological dynamics and patterns of populations and communities (Chave, 2013; Levin, 1992).

### 4.2 Effects of environmental drivers on breeding performance in the two populations

The differential environmental conditions above‐mentioned between the two study localities likely resulted in contrasting responses of females' breeding performance in both populations. Individuals from Montseny living at higher elevations, that is, with colder and wetter climates, and within a much larger patch of deciduous mixed forests, showed a positive association of performance with seed availability and higher population densities than in Montnegre. Previous studies have already shown differential life history tactics between core populations of dormice depending on the homogeneity of seed tree production (Bieber & Ruf, 2009). In mixed deciduous forests, the number of pups produced by females each year was higher than that in homogeneous beech habitats, but considering that adult survival showed the opposite trend, estimated lifetime reproductive success was higher in homogeneous beech habitats (Bieber & Ruf, 2009).

The influence of climate on the number and breeding output of reproductive females was also different in the two study areas: while in Montseny, breeding performance was not affected by weather conditions in spring and summer; in Montnegre, it decreased during dry and hot summers. Interestingly, breeding performance in the Montnegre was not associated with seed availability. Differences between populations also emerged for scores in occupancy parameters (i.e., higher occupancy and colonization, and less extinction probabilities), which were, as expected, higher at Montseny. Our exploration of occupancy dynamics illuminated the pivotal role of deciduous tree cover in predicting the occurrence of breeding females. The positive association between deciduous tree cover and breeding female occupancy suggests the significance of this habitat in shaping the habitat suitability for the edible dormouse (Kryštufek, 2010). Previous studies have shown the intricate plasticity between the temporal dynamics of food availability, phenology, and reproduction in dormice, especially because reproduction and hibernation are crucial and interconnected periods over the lifetime of females (Bieber & Ruf, 2009; Fietz et al., 2005). The notable difference in breeding performance and overall occupancy rates between Montnegre and Montseny further accentuates the habitat‐specific challenges faced by populations at the distribution edge (Usui et al., 2023). We should also consider how trees are responding to environmental changes (climate variability and increased competition during the afforestation process) and how they may modify life histories regarding the production of acorns and nuts and the masting dynamics, especially in their marginal populations (García‐Nogales et al., 2016; Sjölund et al., 2019; Solé‐Medina et al., 2022; Stotz et al., 2021; Wang et al., 2021). Several studies have found that drier conditions in the Mediterranean region may drive a progressive decrease in acorn production and a higher variability over the years (Peñuelas & Sardans, 2021; Sánchez‐Humanes & Espelta, 2011). However, we did not find such a decrease in any of the study populations over the 10 years of study.

The number of reproductive females of the edible dormouse exhibited strong interannual fluctuation but with a higher mean number in Montseny. Our results suggested that in that area, reproduction was influenced by the availability of acorns, but the breeding output (i.e., the number of pups per female) was affected by the availability of beech nuts. This agreed with the relevance of acorns in the diet and reproduction of marginal populations (Vekhnik, 2019), being beech nuts preferred in central‐western Europe (Lebl, Rotter, et al., 2011). Bearing in mind that the availability of beech nuts was lower than that of acorns (in terms of number and biomass), their effects on the breeding performance could be inferred owing to the higher caloric value (7 and 4.4 kcal/g, respectively), and considering that energy‐rich tree seeds are essential for the first winter survival of juveniles (Jurczyszyn, 2018; Lebl et al., 2010). Our results highlight the interplay between the two main seed sources for dormice in Montseny, acorns being a trigger for reproduction (skipped in years with low acorns available), and beech nuts being an additive effect increasing the breeding output. We hypothesized that this was because beech nut production can be predicted with reliability by the presence of buds before the edible dormouse breed.

Despite the above‐mentioned difference between several breeding parameters, the number of pups per breeding female was similar in both populations and within the range of values found in other populations in NE Spain (Míguez et al., 2023). This suggests that density‐dependent processes related to food availability per capita are operating similarly to the breeding performance in our study populations. Density dependence is known to affect dormice habitat use and spatial overlap (Bogdziewicz et al., 2016; Cornils et al., 2017). We note that density dependence is not necessarily stronger in a large population than in a small population, since competition would depend on the ratio between resource and density. A lower dormice density in the Montnegre fits with a lower food and habitat availability. All in all, there is no evidence that the dormice in Montnegre cannot cope with the rising temperature and the small population density. The paleontological record shows that rodents of the extant family Gliridae, to which the edible dormice belongs, have been common in Southern European faunas since the early Eocene when temperatures were much higher than the ones registered in our study (Lu et al., 2021). In Montnegre, the number of females significantly decreased over the study (and consequently, the total number of pups produced), but this can be a demographic resilient mechanism by taking sabbatical years when the environment is harsher, a result that has been found in edible dormice and longer‐lived species (Chambert et al., 2015; Genovart et al., 2018; Pilastro et al., 2003; Ruf et al., 2006; Schlund et al., 2002). Contrarily, skipping reproduction was not recorded in Montseny, likely because the temporal asynchronous availability of both acorns and nuts offered suitable conditions above a threshold value for a physiological response of females for breeding (Fietz et al., 2005; Ruf & Bieber, 2020). Our results confirm previous studies showing that most edible dormouse females would reproduce in seed production years, a lower proportion of the population would reproduce in intermediate seed production years, and most individuals would skip reproduction in years without seed production (Bieber, 1998; Fietz et al., 2005; Lebl et al., 2010; Pilastro et al., 2003; Ruf et al., 2006; Schlund et al., 2002).

At least in core populations, food availability would increase the litter size in dormice in those years with more seed abundance, which would increase annual variability in breeding output (Kager & Fietz, 2009). Nonetheless, our results showed minimal temporal variability in litter size in both study areas, which may be a further local adaptation in marginal populations, where a bet‐hedging strategy could be relaxed compared with what is recorded in core populations (Philippi & Seger, 1989; Stearns, 1976). Previous studies have found that the more marginal population of Montnegre displayed some phenological traits that could be related to local adaptations to these particular environmental conditions (e.g., breeding delayed, pup male‐biased sex ratio; Ferrandiz‐Rovira et al., 2016). Similarly, edible dormice in Montseny likely adapt their life histories to patches with asynchronous seed resources producing lower annual fluctuations in food availability in mixed forests dominated by oaks with interspersed beeches, whereas beech nut production is almost absent in Montnegre (Puig‐Gironès et al., 2023). This suggests that populations in mixed forests may perform differently than in homogenous forests, such as beech‐dominated habitats found in the core dormice populations (Bieber & Ruf, 2009; Cornils et al., 2017; Jurczyszyn, 2018).

Importantly, it is known that dormice may show differences in adult survival over time and between different populations, suggesting ecological plasticity in this parameter (Bieber & Ruf, 2009; Lebl, Bieber, et al., 2011; Ruf et al., 2006). Owing that dormice show an obligate hibernation life history, they have higher adult survival than other rodents of similar size, which indicates that it is a demographic parameter with high sensitivity for their population dynamics. Since we do not know how adult survival changes in the two populations, it is hard to assess whether there is different lifetime reproductive success for females breeding at each locality or in comparison with core populations. Previous studies comparing different populations of dormice show different trade‐offs between reproduction and survival that may be adaptive for optimal life histories (Bieber & Ruf, 2009).

Even though the breeding performance at Montnegre may be showing an adaptation to an increase in temperature and to small population densities, habitat loss by a retraction of deciduous may have ecological, evolutionary, and genetic consequences (Md Naim, 2010; Pironon et al., 2017; Sih et al., 2000). In this small mountain range forests (the potential habitat for dormice would probably cover a single area of about 4 km^2^) with low elevation summits, upward shifts of Eurosiberian deciduous trees are not possible, and they are replaced by perennial sclerophyllous broad‐leaved trees (i.e., *Quercus ilex*) (Ruiz‐Labourdette et al., 2012). Contrarily, populations of dormice in the Montseny, together with other forest species and communities, would potentially spread by the expansion of the deciduous forests (Pereira & Navarro, 2015; Ricotta et al., 2000). The different life history tactics that species or even individuals within populations show in different habitats have received much attention for exploring plasticity and adaptation to cope with the challenges posed by each environment and its stochasticity (Jonsson & Jonsson, 2017; van Noordwijk & de Jong, 1986).

## 5 CONCLUSIONS

Long‐term studies dealing with population responses in marginal habitats can lead to a deeper understanding of the capacities of organisms to adapt to harsh environments and their nonlinear responses, including tipping points for population dynamics (Lynch et al., 2014; Sexton et al., 2009). The demographic characteristics of populations at the edge have the potential to enhance or diminish responses to selection for plasticity via various pathways (Usui et al., 2023). Besides, little is still known about how populations and communities would cope with an increase in extreme climate events that can be frequent in the Mediterranean region, especially at the low‐latitude limit (rear edge) of species distribution (Drake, 2005; Hampe & Petit, 2005; Maxwell et al., 2019; van de Pol et al., 2017; Vautard et al., 2007). For dormice, adaptations and resilient responses have been commonly found and include physiological tolerance of each species for adapting its phenology (i.e., delaying breeding, Ferrandiz‐Rovira et al., 2016), large temporal variability in breeding performance (Schlund et al., 2002; Vekhnik, 2019), the ability to hibernate/estivate for longer periods (Findlay‐Robinson et al., 2023; Oro & Freixas, 2021), the capacity to take sabbatical years for reproduction (Pilastro et al., 2003; Ruf et al., 2006; Schlund et al., 2002), or plasticity to occupy forests with different ecological features, including evergreen trees in Mediterranean edge habitats. Although local adaptation is frequently documented across various taxa, there remains a surprisingly limited comprehension of the underlying mechanisms responsible for its occurrence.

## AUTHOR CONTRIBUTIONS

**Daniel Oro:** Conceptualization (lead); investigation (equal); methodology (supporting); writing – original draft (equal); writing – review and editing (supporting). **Ignasi Torre:** Formal analysis (lead); investigation (supporting); methodology (lead); software (lead); supervision (lead); validation (lead); writing – original draft (equal); writing – review and editing (supporting). **Lídia Freixas:** Conceptualization (lead); data curation (lead); funding acquisition (lead); investigation (lead); methodology (lead); project administration (lead); writing – review and editing (supporting). **Carme Bartrina:** Conceptualization (supporting); data curation (equal); formal analysis (supporting); investigation (supporting); writing – review and editing (supporting). **Silvia Míguez:** Data curation (supporting); investigation (supporting).

## CONFLICT OF INTEREST STATEMENT

None of the authors have a conflict of interest.

## Supporting information


Figure S1.
Table S1.
Table S2.
Table S3.
Table S4.
Table S5.
Table S6.

## Data Availability

Data on dormice are available at CSIC open repository http://hdl.handle.net/10261/361294.
