# Supplementary material for: Direct and indirect effects of climate and seed dynamics on the breeding performance of a seed predator at the distribution edge
Source: Ecol Evol. 2024 Aug 16;14(8):e70104. doi: 10.1002/ece3.70104 (PMC11327614; doi:10.1002/ece3.70104)
Supplement: Supplementary file 1 — Figure S1. Table S1. Table S2. Table S3. Table S4. Table S5. Table S6. [file ECE3-14-e70104-s001.docx]

**SUPPLEMENTARY MATERIAL**

**Figure S1**. Deciduous forest evolution for the period 2007- 2017 in the two study areas. Green dots show the plot sites at the two study populations.


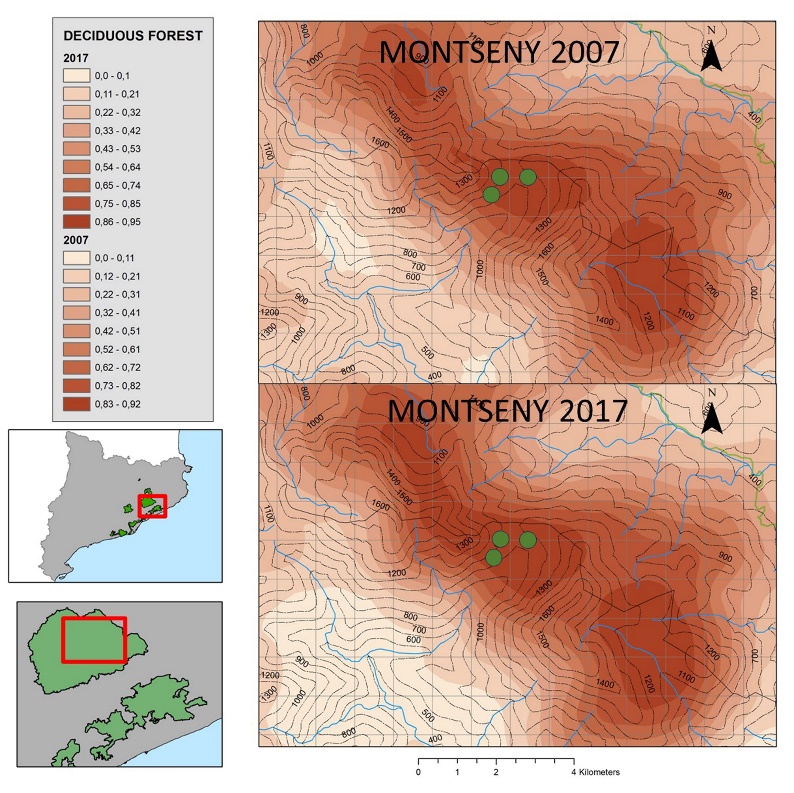

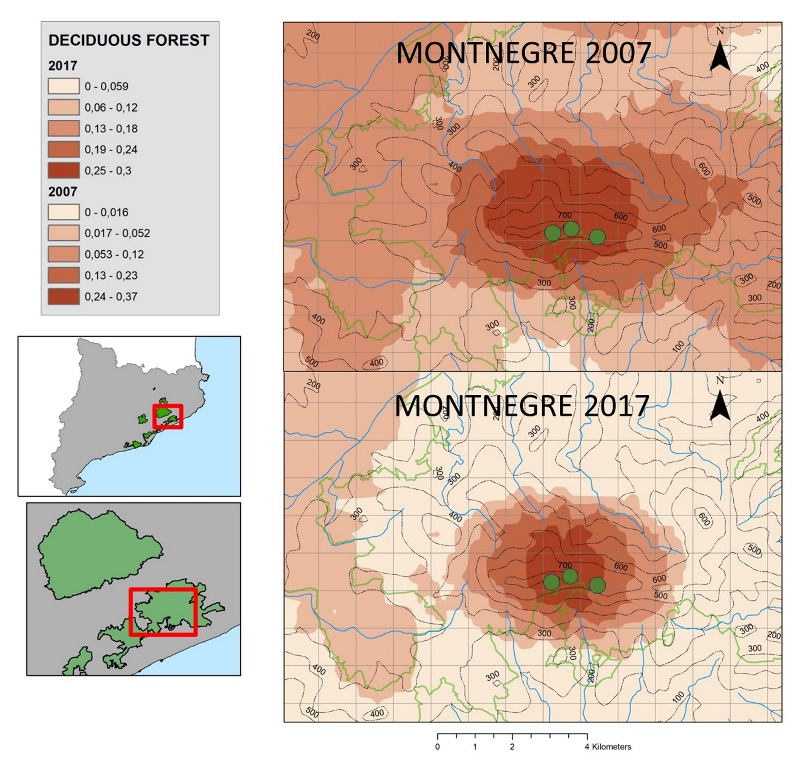


**Table S1.** Mean values (and standard deviations) of seasonal temperature and precipitation in the two studied areas from 2012 to 2021.

| **Year** | **Season** | **Montseny** | | | **Montnegre** | | | |
| --- | --- | --- | --- | --- | --- | --- | --- | --- |
|  |  | Temperature | Precipitation |  |  | Temperature | Precipitation | |
| 2012 | Winter | 4.74 ± 2.67 | 116.50 ± 36.57 |  | 5.77 ± 2.99 | | | 2.80 ± 0.50 |
|  | Spring | 13.42 ± 4.37 | 248.50 ± 42.03 |  | 14.47 ± 3.89 | | | 53.40 ± 9.05 |
|  | Summer | 18.72 ± 2.43 | 168.90 ± 35.49 |  | 19.50 ± 2.09 | | | 77.60 ± 18.80 |
|  | Autumn | 8.26 ±2.84 | 229.30 ± 76.52 |  | 10.30 ± 1.94 | | | 105.00 ± 48.93 |
| 2013 | Winter | 4.45 ± 1.76 | 491.80 ± 177.28 |  | 6.47 ± 1.58 | | | 209.10 ± 58.66 |
|  | Spring | 11.16 ± 2.76 | 304.40 ± 57.21 |  | 12.70 ± 2.65 | | | 141.60 ± 27.84 |
|  | Summer | 17.93 ± 1.77 | 316.30 ± 34.73 |  | 19.53 ± 1.75 | | | 97.00 ± 23.10 |
|  | Autumn | 8.23 ± 3.76 | 246.40 ± 56.32 |  | 10.43 ± 3.55 | | | 158.40 ± 37.68 |
| 2014 | Winter | 5.66 ± 1.01 | 146.90 ± 5.76 |  | 7.70 ± 0.86 | | | 107.60 ± 11.20 |
|  | Spring | 12.89 ± 2.52 | 273.60 ± 18.51 |  | 14.27 ± 2.71 | | | 190.60 ± 13.82 |
|  | Summer | 17.13 ± 0.71 | 306.40 ± 39.96 |  | 17.93 ± 0.80 | | | 157.80 ± 22.40 |
|  | Autumn | 9.07 ± 4.01 | 340.20 ± 111.90 |  | 11.03 ± 3.74 | | | 67.00 ± 21.25 |
| 2015 | Winter | 4.73 ± 1.85 | 189.50 ± 56.46 |  | 6.43 ± 1.76 | | | 104.60 ±10.51 |
|  | Spring | 14.27 ± 3.21 | 120.80 ± 12.27 |  | 15.40 ± 3.40 | | | 91.60 ± 17.63 |
|  | Summer | 18.43 ± 3.33 | 210.60 ± 19.19 |  | 19.20 ± 2.87 | | | 185.60 ± 25.27 |
|  | Autumn | 9.15 ± 1.59 | 234.40 ± 87.35 |  | 11.13 ± 1.48 | | | 97.00 ± 25.97 |
| 2016 | Winter | 5.59 ± 0.07 | 135.80 ± 39.88 |  | 7.50 ± 0.08 | | | 150.20 ± 52.17 |
|  | Spring | 12.18 ± 2.86 | 311.80 ± 40.73 |  | 13.70 ± 3.15 | | | 196.60 ± 37.21 |
|  | Summer | 18.51 ± 1.51 | 95.70 ± 16.73 |  | 20.37 ± 1.04 | | | 98.80 ± 31.81 |
|  | Autumn | 8.68 ± 2.53 | 319.40 ± 23.60 |  | 11.03 ± 2.25 | | | 197.00 ± 47.68 |
| 2017 | Winter | 6.05 ± 2.66 | 313.70 ± 15.63 |  | 8.23 ± 2.42 | | | 24.70 ± 2.42 |
|  | Spring | 14.16 ± 3.98 | 147.30 ± 15.38 |  | 15.47 ± 3.51 | | | 46.40 ± 3.51 |
|  | Summer | 18.10 ± 3.05 | 175.50 ± 37.41 |  | 18.87 ± 2.62 | | | 56.60 ± 2.62 |
|  | Autumn | 7.62 ± 4.11 | 119.20 ± 41.65 |  | 9.63 ± 3.87 | | | 28.90 ± 3.87 |
| 2018 | Winter | 4.15 ± 1.96 | 360.20 ± 47.56 |  | 6.07 ± 2.10 | | | 118.60 ± 17.61 |
|  | Spring | 12.71 ± 2.52 | 386.40 ± 53.97 |  | 14.37 ± 2.50 | | | 204.80 ± 21.67 |
|  | Summer | 18.53 ± 1.40 | 184.60 ± 44.29 |  | 20.30 ± 1.41 | | | 102.20 ± 10.83 |
|  | Autumn | 8.26 ± 2.24 | 641.20 ± 171.89 |  | 10.40 ± 2.05 | | | 388.60 ± 96.27 |
| 2019 | Winter | 5.95 ± 1.94 | 20.00 ± 2.21 |  | 7.87 ± 1.65 | | | 19.20 ± 3.54 |
|  | Spring | 12.03 ± 4.20 | 204.00 ± 29.69 |  | 13.37 ± 3.99 | | | 120.20 ± 10.24 |
|  | Summer | 19.03 ± 2.02 | 204.10 ± 52.62 |  | 20.37 ± 1.74 | | | 93.00 ± 19.41 |
|  | Autumn | 8.16 ± 3.17 | 385.00 ± 54.35 |  | 10.60 ± 2.97 | | | 224.20 ± 18.01 |
| 2020 | Winter | 6.54 ± 1.18 | 526.10 ± 191.22 |  | 8.37 ± 1.14 | | | 209.60 ± 57.20 |
|  | Spring | 12.98 ± 2.48 | 471.30 ± 66.8 |  | 14.83 ± 2.64 | | | 307.20 ± 71.72 |
|  | Summer | 18.00 ± 2.14 | 164.90 ± 12.54 |  | 20.13 ± 1.79 | | | 151.60 ± 19.06 |
|  | Autumn | 7.45 ± 2.52 | 238.20 ± 64.62 |  | 11.65 ± 0.65 | | | 93.00 ± 27.50 |
| 2021 | Winter | 5.30 ± 2.20 | 116.30 ± 21.80 |  | 7.21 ± 4.00 | | | 46.00 ± 4.67 |
|  | Spring | 12.1 ± 4.00 | 131.50 ± 7.50 |  | 13.44 ± 5.35 | | | 106.40 ± 5.11 |
|  | Summer | 18.3 ± 1.50 | 105.70 ± 20.70 |  | 19.86 ± 3.90 | | | 102.80 ± 7.42 |

**Table S2.** GLMMs test the effects of year and locality (and their interaction) on acorn availability, number of breeding females, and number of pups per female.

|  | ACORNS | | FEMALES | | PUPS/FEMALE | |
| --- | --- | --- | --- | --- | --- | --- |
| Fixed effects: | Estimate | SE | Estimate | SE | Estimate | SE |
| (Intercept) | 5.57*** | 0.38 | -0.10 | 0.52 | 6.00*** | 0.94 |
| 2013 | -0.69· | 0.41 | 0.00 | 0.69 | -0.25 | 1.15 |
| 2014 | 1.62*** | 0.41 | 0.00 | 0.69 | -0.25 | 1.15 |
| 2015 | 0.64 | 0.41 | 0.00 | 0.69 | -0.75 | 1.15 |
| 2016 | 0.29 | 0.41 | 0.29 | 0.65 | -0.50 | 1.32 |
| 2017 | 1.57*** | 0.41 | -1.10 | 0.93 | -1.00 | 1.32 |
| 2018 | -0.38 | 0.41 | -0.41 | 0.77 | -2.5· | 1.32 |
| 2019 | -1.46*** | 0.41 | -18.67*** | 1.48 | -3.0* | 1.32 |
| 2020 | -0.72· | 0.42 | -1.10 | 0.95 | 0.00 | 1.32 |
| 2021 | 0.9* | 0.41 | -18.64*** | 1.85 | -3.0* | 1.32 |
| Locality (MSY) | 1.58** | 0.54 | 0.69 | 0.65 | -1.08 | 1.08 |
| 2013:MSY | -1.40* | 0.59 | -0.41 | 0.88 | 2.58· | 1.43 |
| 2014:MSY | -1.03· | 0.58 | 1.25 | 0.77 | 0.76 | 1.38 |
| 2015:MSY | -1.49* | 0.58 | 0.41 | 0.80 | 0.50 | 1.43 |
| 2016:MSY | -1.40* | 0.58 | -0.47 | 0.83 | 2.81· | 1.53 |
| 2017:MSY | -2.07*** | 0.58 | 1.50 | 1.00 | 1.67 | 1.53 |
| 2018:MSY | -1.56** | 0.58 | 0.56 | 0.89 | 3· | 1.58 |
| 2019:MSY | 0.36 | 0.58 | 18.82*** | 1.48 | 3.03· | 1.53 |
| 2020:MSY | -1.12· | 0.58 | 0.69 | 1.07 | 0.58 | 1.53 |
| 2021:MSY | -0.76 | 0.58 | 19.15*** | 1.85 | 3.18· | 1.53 |
| AICc | 884.02 |  | 216.63 |  | 169.47 |  |
| R2marginal | 0.68 |  | 0.97 |  | 0.51 |  |
| R2conditional | 0.82 |  | 0.97 |  | 0.51 |  |
| Residual deviance | 58.05 |  | 15.9 |  | 17.5 |  |
| Ratio | 1.48 |  | 0.84 |  | 0.92 |  |
| dfmodel | 39 |  | 19 |  | 19 |  |
| Model | Negative binomial | | Poisson |  | Gaussian |  |
|  |  |  |  |  |  |  |
| Signif. codes: 0 ‘***’ 0.001 ‘**’ 0.01 ‘*’ 0.05 ‘.’ 0.1 ‘ ’ 1 | | | | |  |  |

**Table S3**. Number of acorns, beech nuts and other seeds production, number of females, pups, and ratio of pups per female (P/F ratio) in the two studied sites from 2012 to 2021.

| **Year** | **Montseny** | | | | | | | | |  | **Montnegre** | | | | | |  |
| --- | --- | --- | --- | --- | --- | --- | --- | --- | --- | --- | --- | --- | --- | --- | --- | --- | --- |
|  | Acorns |  | Beech nuts | Other seeds |  |  | Females | Pups | P/F ratio |  | Acorn | Other seeds | Females | Pups | P/F ratio |  | |
| 2012 | 3707 |  | 36 | 88 |  |  | 8 | 32 | 4.00 |  | 1035 | 63 | 3 | 18 | 6.00 |  | |
| 2013 | 534 |  | 1907 | 895 |  |  | 4 | 29 | 7.25 |  | 465 | 82 | 3 | 17 | 5.67 |  | |
| 2014 | 6947 |  | 1 | 121 |  |  | 22 | 103 | 4.68 |  | 3715 | 40 | 3 | 19 | 6.33 |  | |
| 2015 | 1622 |  | 0 | 188 |  |  | 9 | 44 | 4.89 |  | 1468 | 10 | 5 | 16 | 3.20 |  | |
| 2016 | 1229 |  | 2802 | 573 |  |  | 5 | 35 | 7.00 |  | 1209 | 58 | 4 | 22 | 5.50 |  | |
| 2017 | 2176 |  | 0 | 13 |  |  | 9 | 52 | 5.78 |  | 3468 | 26 | 1 | 5 | 5.00 |  | |
| 2018 | 583 |  | 420 | 661 |  |  | 7 | 40 | 5.71 |  | 831 | 107 | 2 | 7 | 3.50 |  | |
| 2019 | 1334 |  | 24 | 11 |  |  | 7 | 34 | 4.86 |  | 254 | 69 | 0 | 0 | 0.00 |  | |
| 2020 | 633 |  | 1911 | 77 |  |  | 5 | 24 | 4.80 |  | 680 | 36 | 1 | 6 | 6.00 |  | |
| 2021 | 4564 |  | 0 | 35 |  |  | 11 | 52 | 4.72 |  | 1901 | 28 | 0 | 0 | 0.00 |  | |

**Table S4.** Model selection of the SEM tested for the MS population. The selected model is in bold. Params = number of parameters; df = degrees of freedom; C = Discrepancy function; AIC= Akaike Information Criterion; BCC = Browne-Cudeck Criterion; BIC = Bayes Information Criterion. In comparison to the AIC, BCC, and CAIC, the BIC assigns a greater penalty to model complexity and, therefore, has a greater tendency to pick parsimonious models.

| Model | Params | df | C | C - df | AIC | BCC | BIC | C / df | p |
| --- | --- | --- | --- | --- | --- | --- | --- | --- | --- |
| **8** | **21** | **14** | **16.20** | **2.20** | **0.00** | **0.00** | **0.00** | **1.15** | **0.30** |
| 7 | 20 | 15 | 19.92 | 4.92 | 1.72 | 0.96 | 0.32 | 1.32 | 0.18 |
| 9 | 22 | 13 | 14.61 | 1.61 | 0.41 | 1.17 | 1.81 | 1.12 | 0.33 |
| 6 | 19 | 16 | 25.77 | 9.77 | 5.57 | 4.05 | 2.77 | 1.61 | 0.06 |
| 5 | 18 | 17 | 29.50 | 12.50 | 7.30 | 5.01 | 3.09 | 1.73 | 0.03 |
| 10 | 23 | 12 | 13.26 | 1.26 | 1.06 | 2.58 | 3.86 | 1.10 | 0.35 |
| 4 | 17 | 18 | 35.36 | 17.36 | 11.15 | 8.11 | 5.55 | 1.96 | 0.01 |
| 11 | 24 | 11 | 12.24 | 1.24 | 2.04 | 4.32 | 6.24 | 1.11 | 0.35 |
| 3 | 16 | 19 | 41.41 | 22.41 | 15.21 | 11.40 | 8.20 | 2.18 | 0.00 |
| 12 | 25 | 10 | 11.29 | 1.29 | 3.09 | 6.14 | 8.69 | 1.13 | 0.33 |
| 13 | 26 | 9 | 10.53 | 1.53 | 4.33 | 8.14 | 11.34 | 1.17 | 0.31 |
| 14 | 27 | 8 | 9.62 | 1.62 | 5.42 | 9.99 | 13.82 | 1.20 | 0.29 |
| 15 | 28 | 7 | 9.17 | 2.17 | 6.97 | 12.30 | 16.77 | 1.31 | 0.24 |
| 16 | 29 | 6 | 9.16 | 3.16 | 8.96 | 15.06 | 20.17 | 1.52 | 0.16 |
| 2 | 15 | 20 | 58.84 | 38.84 | 30.64 | 26.07 | 22.23 | 2.94 | 0.00 |
| Null 3 | 7 | 28 | 87.71 | 59.71 | 43.51 | 32.84 | 23.89 | 3.13 | 0.00 |
| Null 4 | 8 | 27 | 85.88 | 58.88 | 43.68 | 33.77 | 25.46 | 3.18 | 0.00 |
| Sat | 35 | 0 | 0.00 | 0.00 | 11.79 | 22.46 | 31.41 |  |  |
| 1 | 14 | 21 | 87.71 | 66.71 | 57.51 | 52.17 | 47.70 | 4.17 | 0.00 |
| Null 1 | 14 | 21 | 87.71 | 66.71 | 57.51 | 52.17 | 47.70 | 4.17 | 0.00 |
| Null 2 | 15 | 20 | 85.88 | 65.88 | 57.68 | 53.10 | 49.27 | 4.29 | 0.00 |

Table S5. Model selection of the SEM tested for the MN population. The selected model is in bold. Params = number of parameters; df = degrees of freedom; C = Discrepancy function; AIC= Akaike Information Criterion; BCC = Browne-Cudeck Criterion; BIC = Bayes Information Criterion. In comparison to the AIC, BCC, and CAIC, the BIC assigns a greater penalty to model complexity and, therefore, has a greater tendency to pick parsimonious models.

| Model | Params | df | C | C - df | AIC | BCC | BIC | C / df | p |
| --- | --- | --- | --- | --- | --- | --- | --- | --- | --- |
| 6 | **18** | **9** | **14.66** | **5.66** | **1.27** | **0.64** | **0.00** | **1.63** | **0.10** |
| 7 | 19 | 8 | 11.39 | 3.39 | 0 | 0 | 0.13 | 1.42 | 0.18 |
| 5 | 17 | 10 | 18.93 | 8.93 | 3.54 | 2.27 | 0.86 | 1.89 | 0.04 |
| 8 | 20 | 7 | 10.20 | 3.20 | 0.81 | 1.45 | 2.34 | 1.45 | 0.17 |
| 4 | 16 | 11 | 24.41 | 13.41 | 7.05 | 5.14 | 2.97 | 2.22 | 0.01 |
| 9 | 21 | 6 | 9.70 | 3.70 | 2.31 | 3.58 | 5.24 | 1.61 | 0.14 |
| 3 | 15 | 12 | 31.42 | 19.42 | 12.03 | 9.48 | 6.56 | 2.61 | 0.002 |
| 10 | 22 | 5 | 9.66 | 4.66 | 4.27 | 6.18 | 8.60 | 1.93 | 0.08 |
| 2 | 14 | 13 | 39.21 | 26.20 | 17.81 | 14.63 | 10.94 | 3.01 | 0 |
| Sat | 27 | 0 | 0 | 0 | 4.60 | 9.69 | 15.94 |  |  |
| Null 3 | 6 | 21 | 75.25 | 54.25 | 37.86 | 29.58 | 19.77 | 3.58 | 0 |
| Null 4 | 7 | 20 | 75.18 | 55.18 | 39.78 | 32.15 | 23.10 | 3.75 | 0 |
| Null 1 | 12 | 15 | 75.25 | 60.25 | 49.86 | 45.40 | 40.18 | 5.01 | 0 |
| 1 | 13 | 14 | 74.12 | 60.11 | 50.72 | 46.90 | 42.44 | 5.29 | 0 |
| Null 2 | 13 | 14 | 75.18 | 61.18 | 51.78 | 47.97 | 43.51 | 5.37 | 0 |

**Table S6**. Single-species, Multiple-season occupancy models for the female breeding dormice were ordered according to their statistical fit based on the Akaike Information Criterium (AIC). Model support (AIC weight), Model Likelihood, and number of parameters are also shown (ΔAIC ≤ 2 highlights models with equal support). Ψ: occupancy; γ: colonization; ε: extinction p: detectability. Predictors: cover = deciduous tree cover around sampling plots in 2007; change = deciduous tree cover change around sampling plots between 2007 and 2017; Loc = Locality (Montseny and Montnegre).

| Model | AIC | ΔAIC | AIC weight | Model Likelihood | no.Par. |
| --- | --- | --- | --- | --- | --- |
| *ψ(cover),γ(·),p(·)* | 372.81 | 0.00 | 0.27 | 1.00 | 4 |
| *ψ(cover),γ(cover),p(cover)* | 373.78 | 0.97 | 0.16 | 0.62 | 6 |
| *ψ(cover),γ(change),p(·)* | 374.82 | 2.01 | 0.10 | 0.37 | 5 |
| *ψ(cover),γ(cover),p(·)* | 374.98 | 2.17 | 0.09 | 0.34 | 5 |
| *ψ(change),γ(change),p(change)* | 375.00 | 2.19 | 0.09 | 0.33 | 6 |
| *ψ(change),γ(change),p(·)* | 375.14 | 2.33 | 0.08 | 0.31 | 5 |
| *ψ(cover),γ(change),p(cover)* | 375.96 | 3.15 | 0.06 | 0.21 | 6 |
| *ψ(Loc),γ(·),p(·)* | 376.08 | 3.27 | 0.05 | 0.20 | 4 |
| *ψ(cover),γ(change),p(change)* | 376.15 | 3.34 | 0.05 | 0.19 | 6 |
| *ψ(change),γ(·),p(·)* | 378.05 | 5.24 | 0.02 | 0.07 | 4 |
| *ψchange,γ(change),ε(change),p(change)* | 379.14 | 6.33 | 0.01 | 0.04 | 8 |
| *ψ(·),γ(cover),p(·)* | 380.87 | 8.06 | 0.00 | 0.02 | 4 |
| *ψ(·),ε(cover),p(·)* | 380.87 | 8.06 | 0.00 | 0.02 | 4 |
| *ψ(·),γ(·),p(·)* | 381.68 | 8.87 | 0.00 | 0.01 | 3 |
